# Synthesis, Characterization and Investigation of Optical and Electrical Properties of Polyaniline/Nickel Ferrite Composites

**DOI:** 10.3390/nano13152223

**Published:** 2023-07-31

**Authors:** Priyanka Kolhar, Basavaraja Sannakki, Meenakshi Verma, Prabhakar S.V., Mansoor Alshehri, Nehad Ali Shah

**Affiliations:** 1 Department of Physics, Gulbarga University, Kalaburgi 585106, India; priyankakolhar25@gmail.com (P.K.); sannakki.phy@gmail.com (B.S.); 2 University Centre for Research and Development, Chandigarh University, Gharuan, Mohali 160055, India; 1707meenakshi@gmail.com; 3 Department of Electronics, Maharani’s Science College for Women (Autonomous), Mysore 570005, India; prabhakarsvele@gmail.com; 4 Department of Mathematics, College of Sciences, King Saud University, P.O. Box 2455, Riyadh 11451, Saudi Arabia; 5 Department of Mechanical Engineering, Sejong University, Seoul 05006, Republic of Korea

**Keywords:** nanoferrite, solution combustion, optical property, electrical conductivity

## Abstract

Nickel ferrite nanoparticles are prepared by using a low-temperature self-propagating solution combustion method using urea as fuel. The prepared nickel ferrite nanoparticles were doped with polyaniline in the three different weight ratios of 10%, 30% and 50% by using an in situ polymerization method and by adding ammonium persulfate as an oxidizing agent. The obtained samples were characterized by using XRD, FTIR, SEM and a UV–visible spectrophotometer. XRD examined crystalline peaks of ferrites and amorphous peak of polyaniline and confirmed the formation of the composites. FTIR examined the chemical nature of samples and showed peaks due to polyaniline and the characteristic peaks that were less than 1000 cm^−1^ wavenumber were due to metal–oxygen bond vibrations of ferrites. AC conductivity increased with frequency in all samples and the highest AC conductivity was seen in polyaniline/nickel ferrite 50%. DC conductivity increased in all samples with the temperature showing the semiconducting nature of the samples. Activation energy was evaluated by using Arrhenius plots and there was a decrease in activation energy with the addition of ferrite content. The UV–visible absorption peaks of polyaniline showed shifting in the composites. The optical direct and indirect band gaps were evaluated by plotting Tauc plots and the values of the optical band gap decreased with addition of ferrite in polyaniline and the Urbach energy increased in the samples with 10%, 30% and 50% polyaniline/nickel ferrite composites. The optical properties of these composites with a low band gap can find applications in devices such as solar cells.

## 1. Introduction

A large part of research focuses on the development of composites with desired novel properties which are developed and enhanced for prevalent emerging applications. Polymers form an excellent class of matrix elements which are doped with different dopants to form composites which have potential applications such as in rechargeable batteries, LEDs, the clothing industry, the automobile industry, packaging and insulation applications, substitutes to woods, adhesives, etc. to cite a few [1,2]. Among the polymers, conducting polymers form a special class as they are intrinsically conducting owing to their structure and are widely used in various applications such as in sensors [3], dye adsorption [4], supercapacitor application [5], electrochromic devices [6], etc. to name a few. Polyaniline is the most widely studied conducting polymer owing to its useful properties such as its easy method of preparation and its chemical stability and definitely its conductivity which is almost metallic and is comparable with mesoscopic metals [7] The conductivity of polyaniline can be significantly enhanced by the addition of suitable dopants [8]. When dopants in the nano dimensions are added to conducting polymers, new properties emerge in the composites. The dopants also improve the existing properties of host polymers. Ferrites form an important class of dopants to polymer which combines the conducting properties of polyaniline with the excellent magnetic attributes characteristic of ferrites, which is a very useful combination of physical properties that possesses pressing importance such as for use in microwave absorption and electromagnetic shielding. The utility of spinel ferrites lies in their desired properties like high resistivity and good magnetic properties of high permeability, high saturation magnetization and low hysteresis loss [9]. Nickel ferrite (NiFe_2_O_4_) has an inverse spinel structure where Fe^+3^ ions located at eight tetrahedral sites and sixteen octahedral sites are shared by both Fe^+3^ and Ni^+2^ ions. The properties exhibited by spinel ferrites depend heavily on the synthesis and processing methods and dopants used. Some of the common methods of preparing spinel ferrite are sol–gel combustion [10], the coprecipitation method [11], hydrothermal synthesis [12], microemulsion polymerization [13], solvothermal synthesis [14], ball milling [15], etc. The method of synthesis and experimental conditions decides the particle size and structure of ferrite particles. Nickel ferrite is suitable for various emerging applications such as radar applications [16], bio-medical [17], water remediation [18], photocatalytic applications [19], etc. The optical properties of nanoferrite composites are less studied and yet are important to be analyzed for use in electro-optical applications. Ferrite particles at nano dimensions with high surface-to-volume ratios show noticeable enhancement in the electrical, optical, mechanical and thermal properties in the composites. Ferrites, being magnetic in nature, have important applications and are widely studied as fillers in composites with regards to their magnetic, electric and optical properties [20,21,22,23]. Gheorghiu F et al. studied the PVDF-Ba_12_Fe_28_Ti_15_O_84_ for the electrical, magnetic and piezoelectric properties and showed that permittivity of the composites increased with the addition of ferrite to PVDF [24]. Darwish MA et al. studied the epoxy/ferrite composite for electrical and dielectric properties and found that composites of epoxy/ferrite had higher AC conductivity than that of epoxy resin and also found that a higher amount of ferrite in the composites cause defects and decreased the AC conductivity [25]. Leila Abbasi et al. studied polyaniline/ferrites thin films (lead, nickel and copper ferrite) for the magnetic properties and their applications in electromagnetic shielding materials [26]. Elsayed AH et al. studied the magnetic properties of polyaniline/ferrites and found the composites to be ferromagnetic and also found that the samples showed increased thermal stability with the addition of ferrites to polyaniline [27].

In the present study, the conducting polymer polyaniline was selected for the matrix and to it nano-nickel ferrite was doped in the three wt% of 10%, 30% and 50%. Nickel ferrite is synthesized by using the single-step self-propagating solution combustion method. The composites are prepared by doping ferrite by using the in situ polymerization method, which ensures the homogeneous scattering of nanoferrite particles in the polyaniline grid. XRD, FTIR and SEM analyses are carried out and detailed optical properties and AC/DC conductivity are studied. The less studied optical and electrical properties of these powdered nanocomposite samples are also studied. In terms of optical properties, the direct band gap, the indirect band gap and the Urbach energy are studied, and, in terms of electrical properties, AC/DC conductivity are studied as a function of frequency. The estimation of the optical band gap is needed in order to understand photophysical behavior in semiconductors.

## 2. Methodology

Materials. All chemicals of AR grade were procured from Otto Chemicals (Mumbai).

### 2.1. Synthesis of Nickel Nanoferrite

Iron nitrate nonahydrate and nickel nitrate hexahydrate metal nitrates are taken in the molar ratio of 1:2 in 100 mL deionized water. The oxidizing valencies of metal nitrates are balanced by reducing valencies of the urea and therefore 6.66 moles of urea is added to the mixture of the metal nitrates solution. The solution is stirred well to obtain a clear homogenous solution and place it in a silica crucible. The solution is heated in the muffle furnace for up to 500 °C to obtain fine voluminous nickel ferrite nanopowder which is crushed in agate in a mortar pestle and then calcined for 3 h at 300 °C.

### 2.2. Preparation of Polyaniline/Nickel Ferrite Composites

The obtained nano-NiFe_2_O_4_ powder is added in different percentages (10%, 30% and 50%) during polymerization of aniline. The aniline monomer (0.2 M) is added to HCl in a beaker and constantly stirred with a magnetic stirrer, resulting in the formation of aniline hydrochloride. The nickel ferrite powder in different weight percentages is added to this and further ammonium persulfate, the oxidizing agent, is added drop by drop continuously to the solution till the color of the solution changes from brownish to dark green. The temperature of the solution is maintained between 0 °C and 5 °C by placing it in the beaker with ice cubes. The solution is stirred continuously and further placed for polymerization. Whatman filter paper is used to filter the solution and the resulting precipitate obtained is washed with distilled water and acetone multiple times to remove any impurities. The precipitate is dried in air and then dried in a hot air oven at 60 °C to get polyaniline/nickel ferrite composites of 10%, 30% and 50 wt% as reported in an earlier study [28].

### 2.3. Materials Characterization

The crystal structure identification of the powdered samples is carried out with an X-ray diffractometer (Rigaku miniflex, Cedar Park, TX, USA) with a monochromatic CuK_α_ radiation source in the 2θ range of 10–80° with a scan rate of 4°/min. FTIR is carried out for all the samples by mixing powder with KBr and pressing it to form pellets and then analyzing it with a PerkinElmer (model-1000, perkin Elmer, Waltham, MA, USA) spectrometer in the wavenumber range of 400 to 4000 cm^−1^. SEM analysis was carried with a scanning electron microscope PHENOM PROX for morphological study. To study the AC conductivity of the samples, pellets are prepared with a hydraulic press and silver paste is used to make contacts on either side for electrical measurements. The dielectric data are obtained with a PC-based LCR meter (HIOKI 3532-50 HITESTER, TEquipment, Long Branch, NJ, USA) and AC conductivity is calculated from the dielectric data, as a function of frequency at room temperature, in the frequency range of 50 Hz to 5 MHz. DC conductivity is measured with a Keithley electrometer [Model: 6514] for a temperature range of 45 °C to 180 °C. The optical absorption data are recorded with a double beam monochromatic T90+ UV–visible spectrophotometer in the wavelength range of 200–800 nm at room temperature.

## 3. Results and Discussion

### 3.1. XRD Analysis

**Figure 1:**
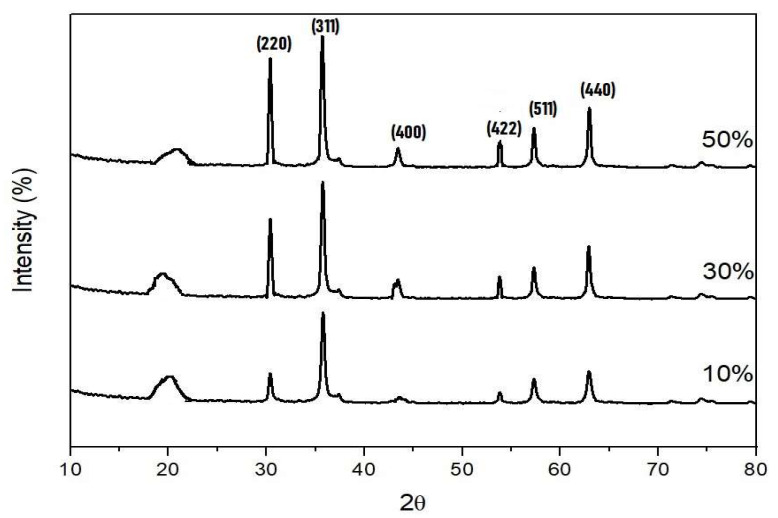
XRD patterns of the polyaniline/nickel ferrite composites.

XRD studies were carried out to understand the nature for phase identification of a crystalline material. The XRD pattern of the different composite is seen in Figure 1. The graph shows both amorphous and crystalline structure with one broad peak of polyaniline around the 2θ angle of 23° and also shows sharp high intensity peaks of nickel ferrites. In the 50% composites, we see sharp crystalline peaks at 2θ angles of 30.5°, 35.6°, 43.5°, 53°, 57.4° and 63°, which correspondingly could be indexed to (hkl) planes of (311), (400), (331), (422) and (511) [29]. As the percentage of ferrite increases in the polyaniline matrix, we see the decrease in the intensity of the polyaniline peak whereas the crystalline peaks of ferrites become more intense in the samples. The crystallite size of composites is calculated using the Debye–Scherrer formula, for the most intense (311) peak, and it takes values of 16 nm, 19 nm and 24 nm for the 10%, 30% and 50% composites, respectively.

### 3.2. SEM Analysis

**Figure 2:**
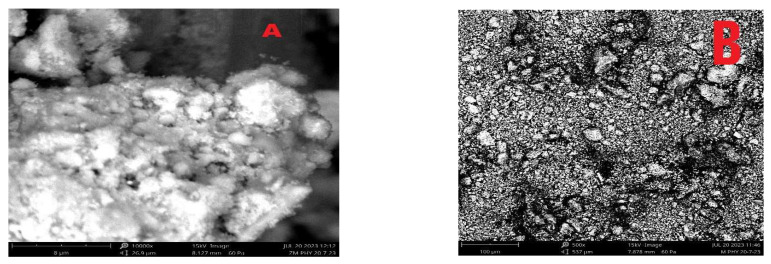

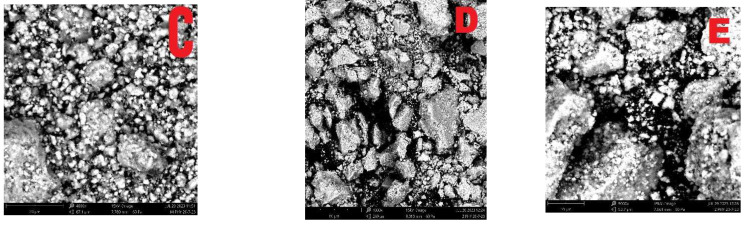
SEM images: (**A**) polyaniline, (**B**) nickel ferrite, (**C**) 10% composite, (**D**) 30% composite, (**E**) 50% composite.

The morphology of the samples of polyaniline, nickel ferrite and polyaniline/nickel ferrite composites of 10%, 30% and 50% were analyzed with SEM images shown in Figure 2. The SEM image of polyaniline shows a fibrous agglomerated structure where the small granules connect to each other to form large grains. Nickel ferrite nanoparticles appear as spherical small particles. The composites of different weight percentages show different morphologies. In the different composites, we see the wrapping of the nickel ferrite nanoparticles in the polyaniline structure [30]. As the percentage of ferrite increases in the composites, we see that there is more interlinking of ferrite particles to the polyaniline structure. The more interconnected structure is linked to increased conductivity in the samples.

### 3.3. FTIR Analysis

**Figure 3:**
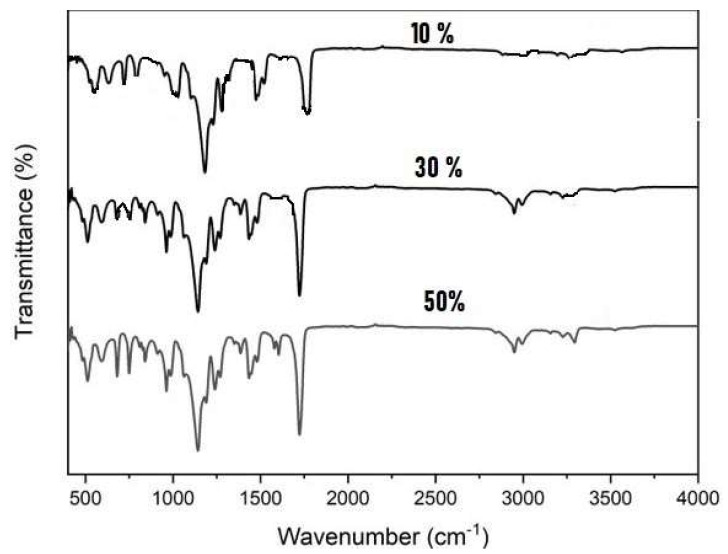
FTIR spectra of polyaniline/nickel ferrite composites.

The chemical composition and functional groups were studied with FTIR. FTIR was carried out for all the samples by mixing powder with KBr and pressing to form pellets and then performing analysis with a PerkinElmer (model-1000) spectrometer in the wavenumber range of 400 to 4000 cm^−1^. The XRD pattern is seen in Figure 3, with peaks due to ferrites and polyaniline. The peaks less than 1000 cm^−1^ are due to ferrites, which occur at 512 cm^−1^ and 591 cm^−1^ and are attributed to the vibrations of metal ion and oxygen bonding at octahedral and tetrahedral sites, respectively, in the spinel structure [31], and FTIR peaks due to polyaniline are explained in Table 1 [32]. In the composites, the wavenumbers shift slightly to lower wavenumbers in the higher percentages of ferrites, which is attributed to the interaction of ferrites with polymer chains [33]. The main characteristic peak positions of PANI were almost same in all the composites, and the differences in intensities could be attributed to differences in the protonation level or oxidation level in polyaniline.

### 3.4. AC and DC Conductivity

The electrical conductivity of the material is given as

(1) $$\sigma \left[\omega ,T\right]=\sigma_{dc}\left[T\right]+\sigma_{ac}\left[\omega ,T\right]$$

*dc* conductivity is temperature-dependent, and *ac* conductivity is a function of temperature. AC conductivity is calculated with dielectric data using the Equation (2):

(2) $$\sigma_{ac}=2\pi \nu \varepsilon^{\prime }\varepsilon_{0}\tan\;\delta \;$$

where *ν* is the frequency, *ε′* is the real part of complex dielectric constant and *ε*_0_ is the permittivity of space; tan δ is the measured tangent loss.

**Figure 4:**
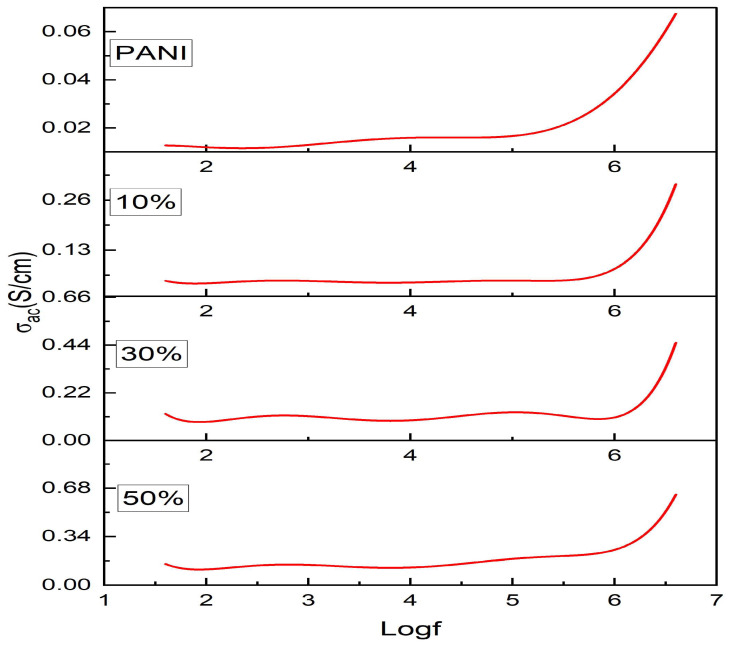
Variation in AC conductivity as a function of frequency.

Figure 4 shows the variation in AC conductivity as a function of frequency and in all the samples, conductivity increases with increasing frequency and the dispersive nature at high frequency is as per the Jonscher universal power law. AC conductivity is almost constant up to 10^5^ Hz and then exponentially increases with frequency, which could be attributed to the contribution from polarons and bipolarons [34]. A plateau region and an exponential increase in AC conductivity is a characteristic of disordered materials in which conductivity is mainly due to hopping of carriers [35]. It is generally known that the conductivity of a composite depends on factors such as the protonation state of polymer and the crystallinity percentage, in addition to frequency and temperature [36]. All composites show higher conductivity than polyaniline and the highest conductivity is seen in the 50% nanocomposite with a value of 0.85 S/cm. The addition of ferrite to PANI facilitates charge transfer in the polymer chain, causing increased conductivity and thus the AC conductivity of polyaniline/nickel ferrite composites can be optimized by suitable doping with nanoferrites.

**Figure 5:**
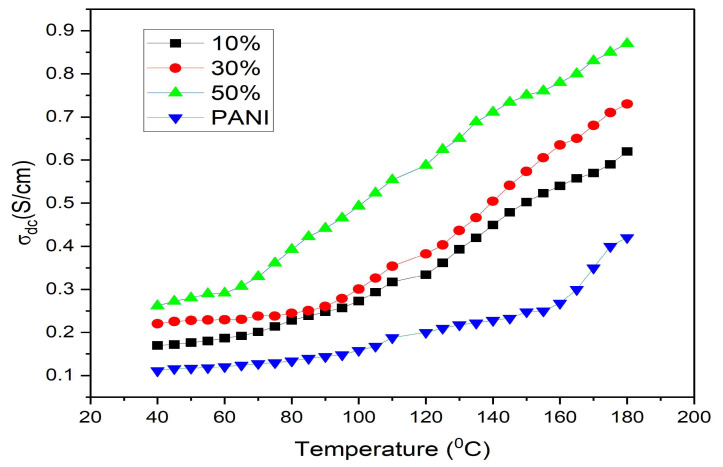
DC conductivity as a function of frequency.

**Figure 6:**
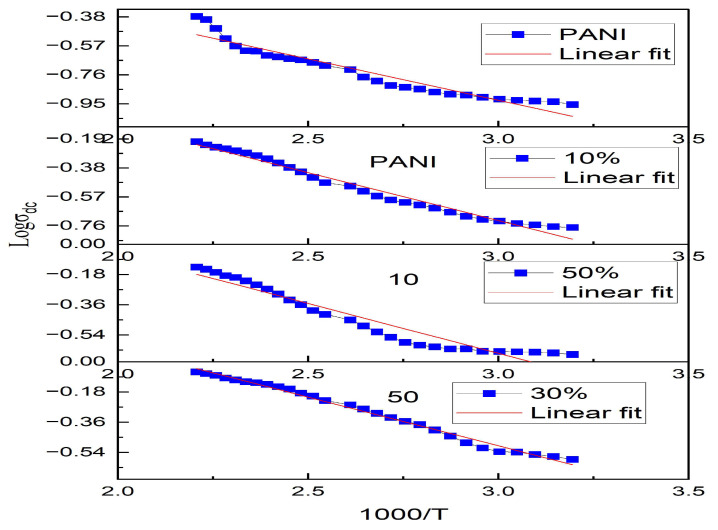
Arrhenius plot log (σ) vs. 1000/T for polyaniline/nickel ferrite composites.

The DC conductivity is measured by using a two-probe method with a Keithley electrometer for all the samples at a temperature range of 40–180 °C as shown in Figure 5. The DC conductivity increased with temperature for all the samples, indicating their semiconductor nature, which could be due to the formation of an organized path network for transportation of charge carriers from the added dopants [37]. The DC conductivity increased in samples with the addition of ferrite up to 30% and decreased with the further addition of ferrite, which could be due to conduction path blocking by the dopant nanoferrite. The DC conductivity nature of the composites dependent on temperature indicates that at higher temperature, the charge carriers gain higher energy to be excited to the conduction band, which is a thermal process best described by the Arrhenius equation given as equation no (3) where $\sigma_{0}\;$ is the constant dependent on the material, *E_a_* is the activation energy, *k_B_* is the Boltzmann constant and *T* is the absolute temperature. Figure 6 shows the Arrhenius plots of ln(*σ_dc_*) vs. (1/*T*) for all the samples of polyaniline/nickel ferrite composites, and it presents the changes seen in DC conductivity as a function of temperature for all the samples of polyaniline/nickel ferrite composites. The slope of the linear fit to the plot is used to evaluate the activation energy. The activation energy of polyaniline is less than that of composites but among the different wt% composites, we see the trend of decreasing activation energy with the addition of higher content of nickel ferrite nanoparticles as dopants. The activation energy calculated for polyaniline and its different wt% composites are listed in Table 2. Henaish AM et al. also found that the activation energy of polyaniline/ferrite decreased with increasing ferrite content [38]. The decreased activation energy among the composites supports the increased electrical conductivity in the composites

(3) $$\;\;\;\sigma =\sigma_{0}\;exp\;exp\;\left(\frac{-E_{a}}{k_{B}T}\right)\;$$

### 3.5. Optical Properties

**Figure 7:**
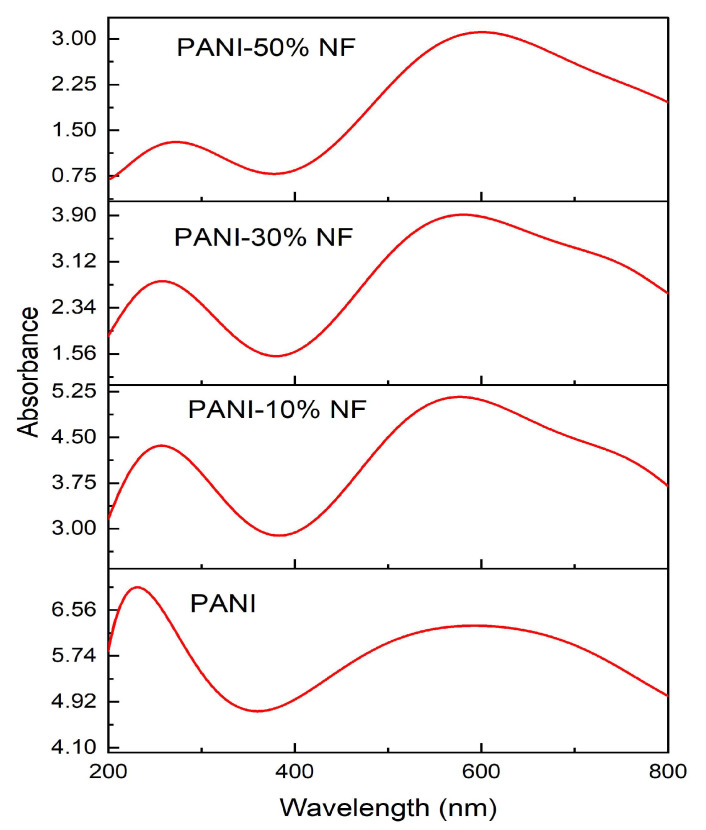
UV–visible absorbance spectra of polyaniline (PANI)–nickel ferrite (NF) composites.

UV–visible absorption was studied to understand the optical properties of polyaniline/nickel ferrite composites. Figure 7 shows the spectra of all the prepared samples in the wavelength range of 200–800 nm. The UV–visible absorbance pattern at room temperature for 10%, 30% and 50% composite was carried out to study the optical band gap variation due to the induced electronic transitions. During photoexcitation, an electron jumps from the valence band to the conduction band across the optical band gap. Polyaniline has two absorption bands at 229 nm and 573 nm. The absorption band at 229 nm is due to the π–π* transition of the phenyl ring. The 573 nm absorption band is present because of the charge transfer from HOMO in the benzenoid ring to LUMO in the quinoid ring [39]. In the composites, the first peak occurs at 280, 294 and 300 nm for 10%, 30% and 50% composites, respectively, showing a shift. The peak at 649 nm in polyaniline appears to be shifted to 568, 590 and 612 nm in the composites of 10%, 30% and 50%, respectively, and is due to the interaction of oxygen in nickel nanoferrites with –NH of polyaniline [40]. The absorption coefficient (*α*) is calculated with Equation (4)

(4) $$\alpha =\frac{2.303A}{T}$$

**Figure 8:**
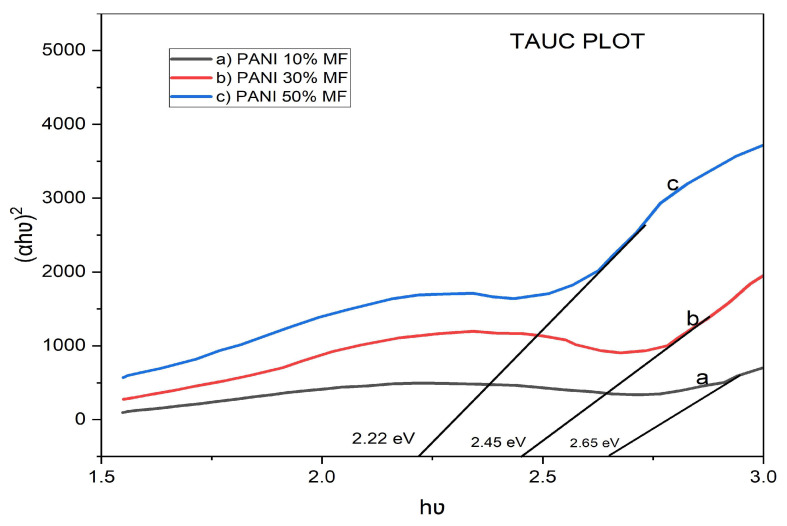
Direct band gaps of polyaniline/nickel ferrite (NF) composites.

**Figure 9:**
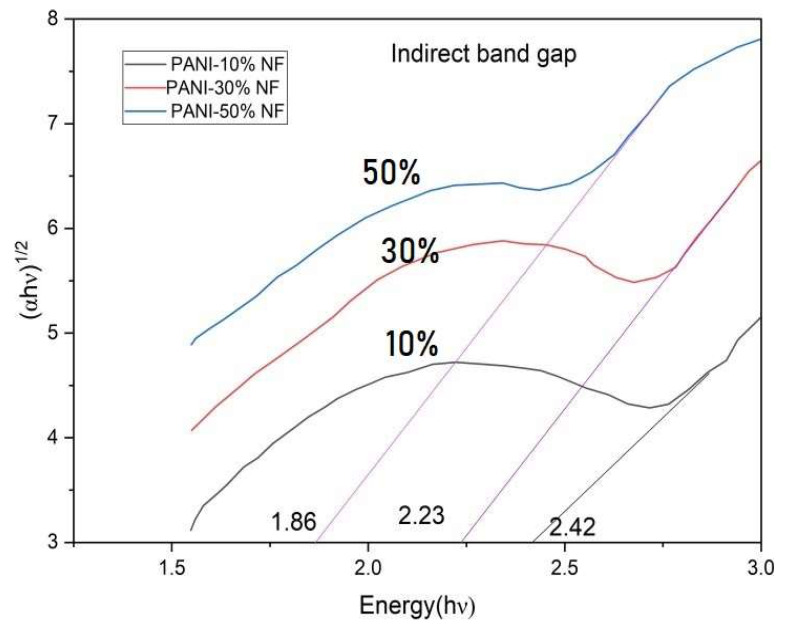
Indirect band gaps of polyaniline/nickel ferrite (NF) composites.

In Equation (4), *A* is the optical absorbance. The thickness of the sample (*T*) is the length of cuvette in which the sample solution (sample dissolved in deionized water) is placed equal to 1 cm. The Tauc equation was used to evaluate the optical direct and indirect band gaps and is given as Equation (5)

(*α*ℎv)^*n*^ = (ℎv − *E_g_*) (5)

where *h* is Planck’s constant, B is a constant dependent on the transition probability, *E_g_* is the optical band gap and *n* is an index which is theoretically taken to be a value of 2 for direct allowed transition and a value of 1/2 for indirect allowed transition. Using this relationship, the direct optical band gap and indirect optical band gap values are calculated for all the composites. For the determination of the direct band gap, the linear region is extrapolated on the x-axis on a graph of (*αh*v)^2^ vs. *h*v shown in Figure 8. For determination of the indirect band gap, the linear region is extrapolated on the x-axis on a graph of (*αh*v)^1/2^ vs. *h*v as seen in Figure 9. The direct optical band gap values calculated from graph are 2.65 eV, 2.45 eV and 2.22 eV for 10%, 30% and 50% polyaniline/nickel ferrite composites and the indirect band gap calculated values are 2.42 eV, 2.23 eV and 1.86 eV for 10%, 30% and 50% polyaniline/nickel ferrite composites. With the addition of nickel ferrite to polyaniline, there is an increase in the defects of composites, creating new levels, and therefore we see the decreasing values of direct and indirect band gaps in the composites. Smita Chaturvedi et al. reported a similar inference for PANI/ferrite composites [41].

### 3.6. Urbach Energy

**Figure 10:**
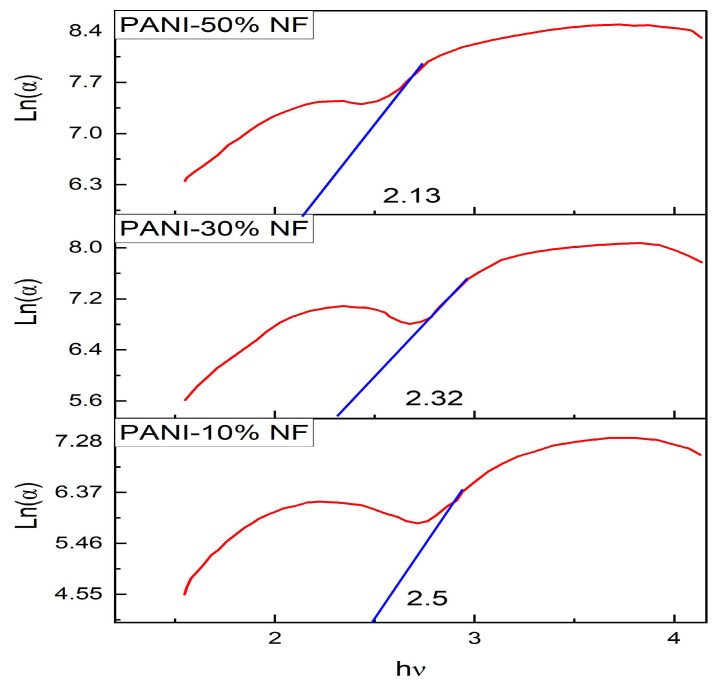
Plot of ln(α) vs. hν of composites.

Urbach energy represents the steepness of optical absorption onset near the band edge. The Urbach rule calculates the exponential dependence of absorption coefficient on the photon energy at the band edge. The optical activation energy of all the composites is determined by reciprocating the slope of the linear part of the plot of ln(*α*) at α = 0 with the photon energy (h*ν*) plotted in Figure 10. The optical energies calculated for the 10%, 30% and 50% polyaniline/nickel ferrite composites are listed in Table 3. The Urbach energy increases as the percentage of nickel ferrite increases in the polyaniline matrix.

## 4. Conclusions

Nickel nanoferrite particles were prepared by using a low-temperature self-propagating solution combustion method and the composites of polyaniline/nickel ferrite were prepared by doping nickel ferrite nanoparticles in different weight percentages during the polymerization of aniline. The XRD of polyaniline/nickel ferrite 50% showed the broad peak of polyaniline, confirming the amorphous nature of the polymer and the XRD spectra also displayed sharp characteristic peaks of nickel ferrite. The SEM images showed a highly agglomerated structure of polyaniline and spherical particle structure for ferrite particles. FTIR confirmed the formation of the composite and displayed peaks of polyaniline and the peaks that were less than 1000 cm^−1^ corresponded to metal–oxygen vibrations of ferrites. The AC and DC conductivity in all composites were higher than that of polyaniline. AC conductivity at room temperature increased as a function of frequency and was highest for the 50% composite. The DC conductivity confirmed the semiconducting nature of the samples, as we see the increase of DC conductivity as a function of temperature. The activation energy decreased with the addition of higher wt% of nickel ferrite. Thus, the needed level of electrical conductivity can easily be achieved by employing the required wt% of polyaniline and nickel ferrite. The UV absorption peaks of polyaniline showed shifting in the composites. As the content of ferrite increased in polyaniline, the optical direct and indirect band gap values decreased. The Urbach energy values increased with the increasing percentage of ferrite in the polyaniline matrix.

## Figures and Tables

**Table 1:** FTIR peak assignments of polyaniline/nickel ferrite composites.

Peak Wavenumber (cm^−1^)	Assignment of Peak
1577	Non-symmetric vibration of C=C bond of quinoid ring
1440	Non-symmetric vibration of C=C bond of benzenoid ring
1270	C–N bond stretching in quinoid
1238	C–N bond stretching in benzenoid
1146	C–H bending in plane
840	C–H out-of-plane bending
684	Aromatic C–H out-of-plane bending vibrations in benzene ring
2900–3200	N–H vibrations of secondary amine

**Table 2:** Activation energy of polyaniline (PANI)–nickel ferrite (NF) composites.

Sl.No	Name of the Sample	Activation Energy (*E_a_*) eV
1	PANI	0.043
2	PANI-NF 10%	0.050
3	PANI-NF 30%	0.046
4	PANI-NF 50%	0.051

**Table 3:** The direct band gap, indirect band gap and Urbach energies of the polyaniline/nickel ferrite composites.

S. No	Name of the Samples	Direct Band Gap (eV)	Indirect Band Gap (eV)	Urbach Energy (eV)
1	PANI-10% NF	2.65	2.42	0.46
2	PANI-30% NF	2.45	2.23	0.43
3	PANI-50% NF	2.22	1.86	0.40

## Data Availability

Data available on request.
